# Gene losses, parallel evolution and heightened expression confer adaptations to dedicated cleaning behaviour

**DOI:** 10.1186/s12915-023-01682-3

**Published:** 2023-08-23

**Authors:** Jingliang Kang, Sandra Ramirez-Calero, José Ricardo Paula, Yifang Chen, Celia Schunter

**Affiliations:** 1 Swire Institute of Marine Science, School of Biological Sciences, The University of Hong Kong, Pokfulam, Hong Kong SAR, China; 2 MARE – Marine and Environmental Sciences Centre & ARNET – Aquatic Research Network, Laboratório Marítimo da Guia, Faculdade de Ciências, Universidade de Lisboa, Av. Nossa Senhora Do Cabo, 939, Cascais, 2750-374 Portugal; 3 Departamento de Biologia Animal, Faculdade de Ciências, Universidade de Lisboa, Campo Grande, 1749-016 Lisbon, Portugal; 4 State Key Laboratory of Marine Pollution and Department of Chemistry, City University of Hong Kong, Hong Kong, SAR, China

**Keywords:** Gene family contraction, Sensory receptors, Immune system, Neural signal transduction, *Labroides dimidiatus*

## Abstract

**Background:**

Cleaning symbioses are captivating interspecific interactions in which a cleaner fish removes ectoparasites from its client, contributing to the health and diversity of natural fish communities and aquaculture systems. However, the genetic signatures underlying this specialized behaviour remain poorly explored. To shed light on this, we generated a high-quality chromosome-scale genome of the bluestreak cleaner wrasse *Labroides dimidiatus*, a dedicated cleaner with cleaning as primary feeding mechanism throughout its life.

**Results:**

Compared with facultative and non-cleaner wrasses, *L. dimidiatus* was found with notable contractions in olfactory receptors implying their limited importance in dedicated cleaning. Instead, given its distinct tactile pre-conflict strategies, *L. dimidiatus* may rely more heavily on touch sensory perception, with heightened gene expression in the brain in anticipation of cleaning. Additionally, a reduction in NLR family CARD domain-containing protein 3 might enhance innate immunity of *L. dimidiatus*, probably assisting to reduce the impacts from parasite infections. In addition, convergent substitutions for a taste receptor and bone development genes across cleaners (*L. dimidiatus* and facultative cleaners) may provide them with evolved food discrimination abilities and jaw morphology that differentiate them from non-cleaners. Moreover, *L. dimidiatus* may exhibit specialized neural signal transductions for cleaning, as evidenced by positive selection in genes related to the glutamatergic synapse pathway. Interestingly, numerous glutamate receptors also demonstrated significantly higher expression in *L. dimidiatus* not engaged in cleaning, as compared to those involved in cleaning. Besides, apparent contractions in *L. dimidiatus* for protocadherins, which are responsible for neuronal development, may further promote specialized neural signal transductions in this species.

**Conclusions:**

This study reveals that *L. dimidiatus* harbours substantial losses in specific gene families, convergent evolutions across cleaners and a large-scale high gene expression in preparation for cleaning, allowing for adaptation to the dedicated cleaning behaviour.

**Supplementary Information:**

The online version contains supplementary material available at 10.1186/s12915-023-01682-3.

## Background

Cleaning symbioses are cooperative interspecific interactions between a cleaner and a usually larger client, where a cleaner removes and consumes materials that can negatively impact a client. These cleaning interactions are widespread among marine animals, and a total of 208 fish and 51 shrimp species have so far been described as cleaners [1]. A cleaner fish can ingest a variety of food items including ectoparasites, mucus, injured tissue, or other particles from many client fish species [2, 3]. These cleaning interactions provide benefits for both partners: the cleaner fish receives food, while the client experiences a reduction in ectoparasite load which may otherwise lead to disease, growth reduction and diminished reproductive success [4]. Cleaner fish have been experimentally proven to directly influence fish abundance and species richness in coral reef fish communities [5, 6]. Moreover, they have been shown to enhance aquaculture productions by serving as a biological control against ectoparasites [7, 8].

According to the persistence of cleaning behaviour throughout their life stages, cleaner fishes can be categorized as either dedicated or facultative cleaners. Dedicated cleaners are specialized in feeding almost exclusively by cleaning throughout their entire lives. In contrast, facultative cleaners rely only in small part on cleaning as a food source and/or perform cleaning only during juvenile period [1, 9]. Among 208 reported cleaner fish species, the majority (93%) are facultative cleaners, while only 16 species are dedicated cleaners that depend on cleaning for nearly all of their food [1, 4]. Cleaning behaviours in fish are restricted to the two fish families, the Gobiidae (gobies: 9 dedicated and 5 facultative cleaners) and Labridae (wrasses: 6 dedicated and 62 facultative cleaners) [1].

Several distinct behavioural and morphological characteristics have been proposed as contributions for adaptations to cleaning, such as client attraction methods, colouration and mouth morphology. To initiate cleaning interactions, some cleaners, such as the bluestreak cleaner wrasse *Labroides dimidiatus*, normally stay in their territories called cleaning stations and perform a “dance”, a vertical zig-zag swimming pattern, to attract clients [10, 11]. Furthermore, the striking colouration of cleaner fish, characterized by vivid lateral stripes (typically blue or yellow), enhances their visibility and effortlessly attracts clients for cleaning [4, 12]. In addition, a subterminal mouth and small body size of cleaners may enable them to effectively remove ectoparasites from larger clients [13, 14]. Limited molecular studies on cleaner fish indicate that immediate early genes, glutamate receptors and neurohormones exhibit significant expression changes in brain regions of *L. dimidiatus* during cleaning interactions, and hence play critical roles in cleaning behaviours [15]. Despite parasites appearing as tiny dark dot on clients’ surface and potentially transfer to cleaners causing infections during close-contact cleaning [16, 17], it remains unknow how cleaners can detect parasites efficiently and minimize the infection risk.

The bluestreak cleaner wrasse *L. dimidiatus*, intensively studied for its cleaning behaviour, can interact with over 100 reef fish species [3] and have over 2000 interactions with client fish individuals each day [18], including large predators [12, 19]. However, a major conflict is present since *L. dimidiatus* prefer to eat the protective mucus from their clients, which constitutes cheating [20, 21]. This is the likely cause for the evolution of highly sophisticated cognition and decision rules used by cleaners during interactions. Thus, *L. dimidiatus* are used as a prime example of sophisticated fish cognition, due to their capacity to display self-recognition [22, 23], remember their previous interactions with clients [24], social tool use [25], social learning [26] and reputation management [27]. To investigate the genetic basis underlying these behavioural adaptations needed to perform cleaning, we assembled a high-quality chromosome-level genome of *L. dimidiatus*. By comparing *L. dimidiatus* genome to genomes of seven other Labridae species including five facultative cleaners (*Thalassoma bifasciatum*, *Symphodus melops, Tautogolabrus adspersus, Semicossyphus pulcher, Labrus bergylta*) [1] and two non-cleaners (*Cheilinus undulatus, Notolabrus celidotus*), we examine gene family size, positive selections and convergences, as well as transcriptional regulation for dedicated cleaner *L. dimidiatus*, to pinpoint the molecular adaptations and convergent genomic features underlying the essential cleaning behaviour.

## Results

### Genome sequencing assembly and annotation

For the de novo genome assembly of *L. dimidiatus*, 22.6 gigabase (Gb) PacBio CCS (HiFi) reads (approximately 23-fold coverage) were generated. After removing haplotigs and contig overlaps, the assembly length is 726.36 megabases (Mb) with 256 scaffolds and N50 length of 10.36 Mb. 42.2 Gb of Omni-C (Dovetail) data corresponding to approximately 42-fold coverage were integrated for a final 726.38 Mb chromosome-scale assembly with 57 scaffolds and an N50 of 33.59 Mb (Additional file 1: Table S1), and the 24 largest scaffolds containing 92% of protein-coding genes were deemed as chromosomes (Additional file 2: Fig. S1; Additional file 1: Table S2). The *L. dimidiatus* genome was annotated based on OrthoDB proteins and RNA-seq data, which predicted 37,023 and 61,565 genes respectively. By combining the two predictions, the final annotations indicated 28,138 genes, of which 23,551 (83.7%) genes showed homology with the proteins in Swiss-Prot or vertebrate_other (2021–09-12) in RefSeq. BUSCO analysis [28] on 28,138 predicted genes in *L. dimidiatus* and its genome assembly, revealed that 94.9% (4,349) and 96.7% (4,430) conserved and complete genes were detected using 4584 Actinopterygii genes as reference (Additional file 1: Table S3). Along the *L. dimidiatus* genome, around 35.53% (258.08 Mb) is repeat content, and 27.35% (198.67 Mb) is composed of transposable elements (Additional file 1: Table S4).

### Dynamics of gene family size and evolution of Labridae fish species

**Fig. 1:**
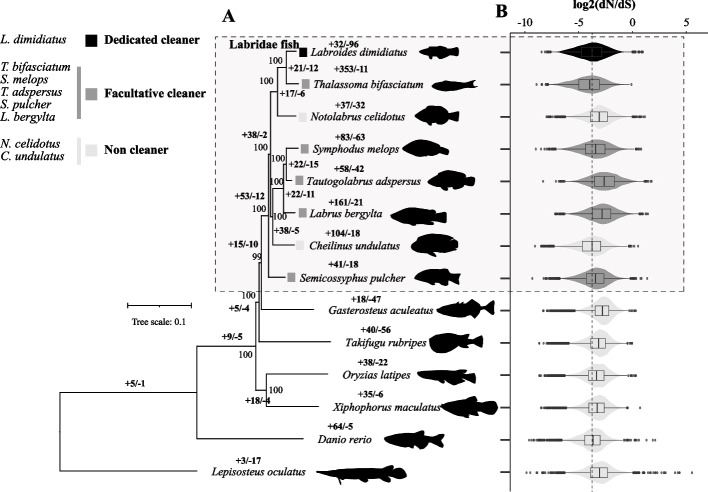
Gene family size change and dN/dS ratios of Labridae fish species. **A** Phylogeny of fourteen fish species including eight Labridae fish species and six reference fish species in this study. Bold numbers indicated the significant expanded and contracted gene families compared to the node of the most recent common ancestor. **B** The dN/dS ratios of eight Labridae and six reference fish species based on 6929 sub-orthogroup genes. *Y* axis indicates the dN/dS ratios of genes per species. The dash line means the median dN/dS of *L. dimidiatus*

Of 22,528 orthogroups among fourteen Labridae fish species with available reference genomes (Fig. 1), 13,826 conserved gene families were retained for investigating the dynamics of gene family size. The eight Labridae fish species diverged ~ 75.29 Million years ago (Mya) and shared a most recent common ancestor with stickleback around 92.17 Mya (Additional file 2: Fig. S2) based on the phylogenetic tree constructed with 2915 single-copy genes (Fig. 1A). Among eight Labridae fish species, *L. dimidiatus* as the only dedicated cleaner fish exhibited 96 contracted gene families (Additional file 1: Table S5) and 32 expanded gene families. The contractions in *L. dimidiatus* were more notable when compared to its two closely related fishes, a facultative cleaner *T. bifasciatum* and a non-cleaner fish *N. celidotus*, such as olfactory receptors (ORs), NLR family CARD domain-containing protein 3 and protocadherins (Additional file 1: Table S5).

Among eight Labridae fishes, the dedicated cleaner *L. dimidiatus* might experience a stronger purifying selection with a relatively lower dN/dS (nonsynonymous-synonymous substitution ratio, mean dN/dS = 0.1106, Fig. 1B], which was only higher than one non-cleaner species *C. undulatus* (mean dN/dS = 0.0986, Wilcoxon rank sum test, *p* = 0.1407) and one facultative cleaner *T. bifasciatum* (mean dN/dS = 0.0944, *p* < 2.2e − 16).

#### Sensory receptor genes

**Fig. 2:**
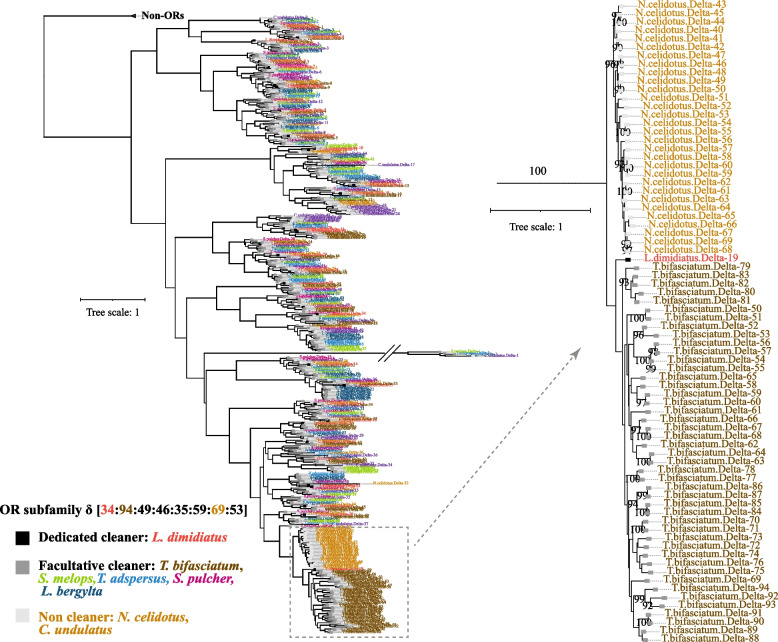
Contractions of olfactory receptors (ORs) in *L. dimidiatus*. Phylogenetic tree of ORs subfamily δ gene sequences with 100 bootstraps and the non-ORs as the outgroup. *T. bifasciatum* and *N. celidotus* exhibited more ORs δ gene than *L. dimidiatus*. The leaf nodes with back, grey and light grey square mean obligate cleaner, facultative cleaner and non-cleaner. The bold branches in the left phylogenetic tree indicate the internal nodes with bootstraps ≥ 80, and only the internal nodes with bootstraps ≥ 80 showed in the right phylogenetic tree

As a main food of cleaner fish, gnathiid isopods are ectoparasites that appear as small dark dots on clients [29]. Olfaction and vision might be critical for cleaner fish to locate the parasites efficiently. Hence, we test for changes in copies of olfactory receptors (ORs) and vision opsin genes. The dedicated cleaner *L. dimidiatus* has 46 ORs, a minimum number among eight Labridae fish species (Additional file 1: Table S6). Compared with two closely related fish species, the facultative cleaner *T. bifasciatum* and the non-cleaner: *N. celidotus*, with 121 and 101 ORs, respectively. The number of subfamilies δ and ζ are contracted in *L. dimidiatus*, with 34 δ and 3 ζ ORs (Fig. 2, Additional file 2: Fig. S3). The facultative cleaner *T. bifasciatum* has 94 δ and 10 ζ OR s, while the non-cleaner *N. celidotus* has 69 δ and 17 ζ ORs. In addition, we examined the visual opsin genes across dedicated, facultative cleaners and non-cleaners for a potential divergence in visual senses. Surprisingly, a non-cleaner *C. undulatus* possessed 16 opsins (Additional file 2: Fig. S4), the largest number among all Labridae species, which was also reported in a previous study [30]. Asides from *C. undulatus*, the other Labridae species displayed a similar number of opsin genes. The only differences between *L. dimidiatus* and its two closely related species (*T. bifasciatum*, *N. celidotus*) lie in the number of rhodopsin 2 (*RH2*). *L. dimidiatus* exhibited three *RH2*, whereas *T. bifasciatum* and *N. celidotus* possess four *RH2*. In addition to olfactory receptors and opsin genes, we also examined the gene number of glutamate receptors (GluRs), comprising ionotropic (iGluRs) and metabotropic (mGluRs) types, which were found to be similar between cleaner and non-cleaner species (Additional file 1: Table S7). Regarding iGluRs, the non-cleaner *N. celidotus* has the fewest number (19 genes), while others have 23–26. As for mGluRs, the eight Labridae species exhibited 9–19 genes, with *L. dimidiatus* (14 genes) similar to facultative cleaners (*S. pulcher*: 13, *T. adspersus*: 14) and non-cleaners (*C. undulatus*: 15, *N. celidotus*: 14).

### Innate immune system

**Fig. 3:**
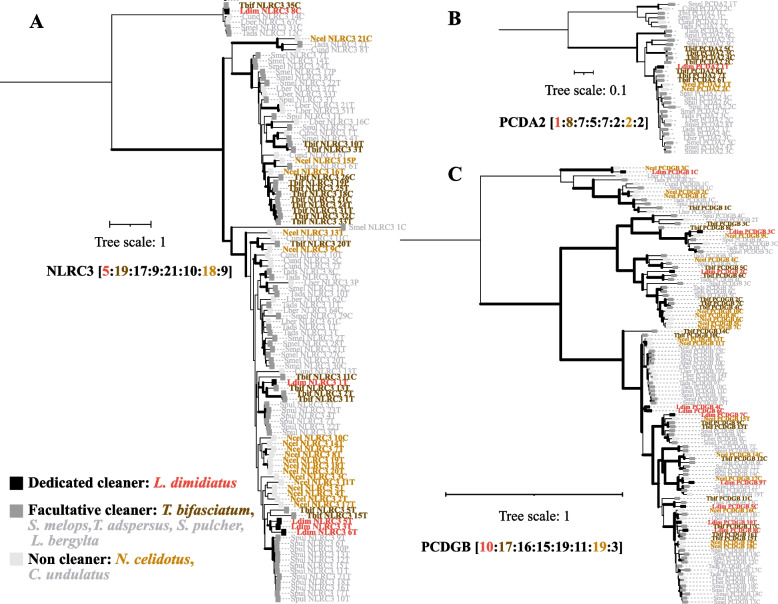
Contractions of *NLRC3* (NLR family CARD domain-containing protein 3) and two protocadherins (*PCDA2*: protocadherin alpha-2; *PCDGB*: protocadherin gamma-A11) in *L. dimidiatus*. Phylogenetic trees of *NLRC3* (**A**), *PCDA2* (**B**) and *PCDGB* (**C**) were constructed by 100 bootstraps and rooting at the midpoint, the leaf nodes with back, grey and light grey square mean obligate cleaner, facultative cleaner and non-cleaner, and the number of each gene showed according to the order as dedicated (*L. dimidiatus*), facultative (*T. bifasciatum*, *S. melops, T. adspersus, S. pulcher, L. bergylta*) and non-cleaners (*C. undulatus, N. celidotus*). The bold branches indicate the internal nodes with bootstraps ≥ 80

Pathogen recognition receptors (PRRs) are critical for the detection of pathogens to initiate innate immune defense [31]. Here we investigated the repertoires of three major PRR families: RIG-like receptors (RLRs), NOD-like receptors (NLRs) and Toll-like receptors (TLRs). *L. dimidiatus* has 31 PRRs (2 RLRs, 16 NLRs, 13 TLRs; Additional file 2: Fig. S5), while *T. bifasciatum* and *N. celidotus* have 52 (3 RLRs, 33 NLRs, 16 TLRs) and 42 PRRs (2 RLRs, 29 NLRs, 11 TLRs), respectively. In particular, *L. dimidiatus* displayed contractions in a NLR gene (*NLRC3*: NLR family CARD domain-containing protein 3) with a minimum number (five copies of *NLRC3*) among eight Labridae fish species (Fig. 3A), and *T. bifasciatum* and *N. celidotus* have 19 and 18 respectively.

### Protocadherins

Protocadherins are homophilic cell adhesion molecules required for neuronal development and synaptic specificity [32]. However, the dedicated cleaner *L. dimidiatus* may have experienced contractions in protocadherins, notable for the alpha and gamma subunits. The dedicated cleaner *L. dimidiatus* genome encodes 22 protocadherin α (Additional file 2: Fig. S6) and 32 protocadherin γ genes (Additional file 2: Fig. S7), while facultative cleaner *T. bifasciatum* and non-cleaner *N. celidotus* have 34 α and 35 γ genes, and 25 α and 41 γ genes respectively. The contractions in *L. dimidiatus* were most notable for an alpha (protocadherin alpha-2: *PCDA2*, Fig. 3B) and a gamma subunit (protocadherin gamma-A11: *PCDGB*, Fig. 3C). Among eight Labridae fish species, *L. dimidiatus* exhibited a minimum number of *PCDA2* (one *PCDA2*) and the second minimum number of *PCDGB* (ten *PCDGB*), while *T. bifasciatum* has eight *PCDA2* and 17 *PCDGB*, and *N. celidotus* has two *PCDA2* and 19 *PCDGB*.

### Estimation of positive selection and convergence

**Fig. 4:**
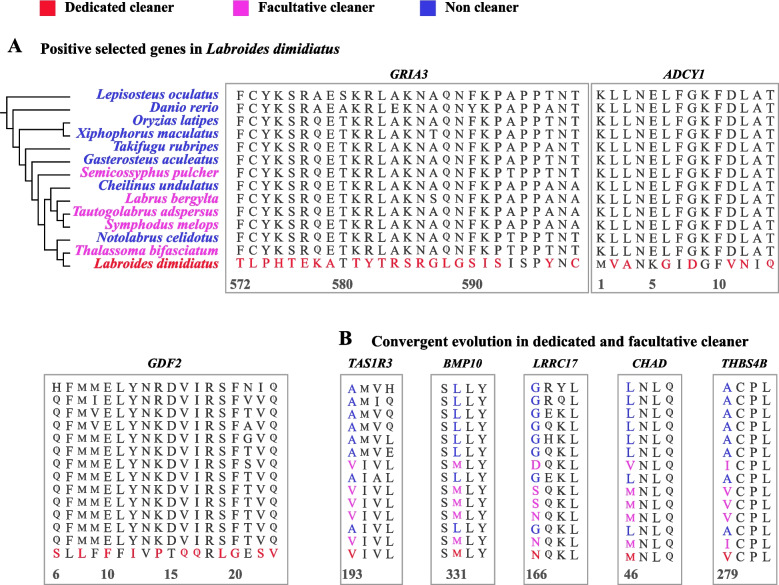
Multi-sequence alignment around substitutions in four positively selected genes of *L. dimidiatus* and convergent evolution in dedicated and facultative cleaners. **A** Unique substitutions of four positively selected genes (*GRIA3*: glutamate receptor 3; *ADCY1*: adenylate cyclase type 1; *GDF2*: growth/differentiation factor 2) in *L. dimidiatus*. The positively selected sites were adopted based on the results from BUSTED-MH in HYPHY. **B** Convergent evolution in four genes (*TAS1R3*: Taste receptor type 1 member 3; *BMP10*: Bone morphogenetic protein 10; *LRRC17*: leucine-rich repeat-containing protein 17; *CHAD*: chondroadherin; *THBS4B*: thrombospondin-4-B) of dedicated and facultative cleaners. All of the cleaners dedicated and facultative cleaners showed convergent amino acid substitutions which were different with the non-cleaners including two Labridae fishes and all of the six reference species

The dedicated cleaner *L. dimidiatus* exhibits 162 positively selected genes (PSGs) identified by PAML [33]. Accounting for the impacts of multi-nucleotide substitutions on natural selection detection, BUSTED-MH in HYPHY [34] was also applied to detect the PSGs, which indicated 45 genes were under positive selection. The 41 genes detected as PSGs by both PAML and HYPHY were considered as the final PSGs (Additional file 1: Table S8). Among these PSGs, it is noteworthy that glutamate receptor 3 (*GRIA3*) and adenylate cyclase type 1 (*ADCY1*) are linked to glutamatergic synapse pathway (Fig. 4A), growth/differentiation factor 2 (*GDF2*) also known as bone morphogenetic protein 9 (*BMP9*), plays a role in regulating cartilage and bone development.

We detected convergence at conservative sites (CCS) [35] to estimate genomic convergences among cleaners. The simulation of amino acid sequences of fish species in the phylogeny of this study (Fig. 4A) indicated that the CCS method reduced random convergences and false convergences by 93.3% (from 10,277 to 687) and 100% (from 1040 to 0, Additional file 1: Table S9). To further remove random convergences, only the genes with CCS in all non-cleaners but not detected in any cleaners were treated as convergent evolving genes (CEGs) in cleaners, which revealed 38 parallel amino acid residue substitutions in 38 genes (Additional file 1: Table S10) across all cleaners (including dedicated and facultative cleaners). Among 38 CEGs, taste receptor type 1 member 3 (*TAS1R3*) is associated with taste sensory perception, and four genes (bone morphogenetic protein: *BMP10*; leucine-rich repeat-containing protein 17: *LRRC17*; chondroadherin: *CHAD*; thrombospondin-4-B: *THBS4B*) are involved in bone morphogenesis (Fig. 4B).

### Cleaning behaviour gene expression in brain regions

**Fig. 5:**
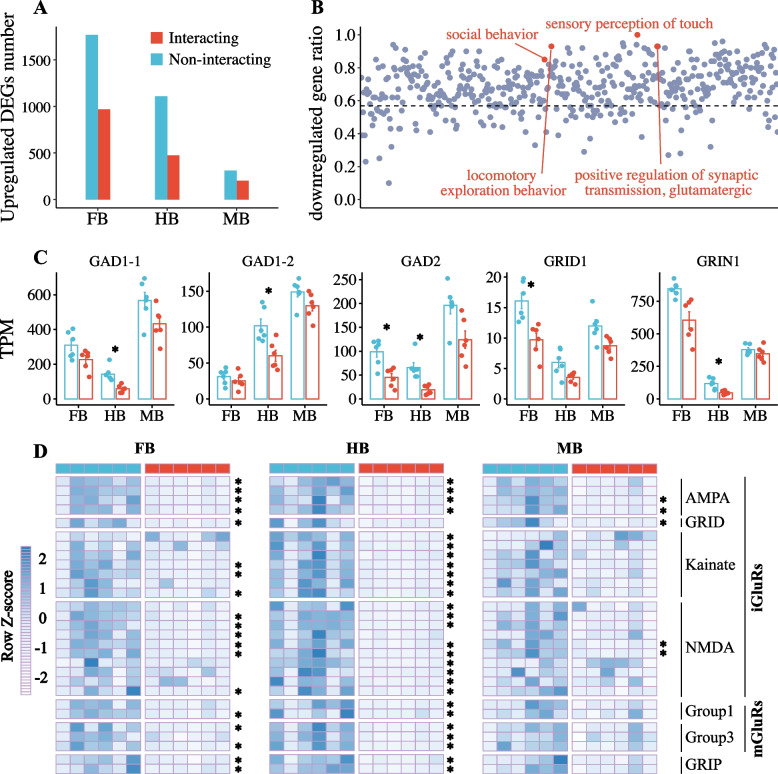
Differentially expressed genes (DEGs) in the forebrain (FB), midbrain (MB) and hindbrain (HB) of *L. dimidiatus* between no-interacting and interacting individuals. Asterisk indicates the brain region with significantly different expression between non-interacting and interacting *L. dimidiatus*. **A** The number of upregulated genes in interacting and non-interacting *L. dimidiatus*. **B** The ratio of downregulated genes in all significantly enriched functions. Here, the downregulated genes indicated genes with reduced expression across all tissues of interacting *L. dimidiatus*. The grey line indicated the downregulated gene ratio of all DEGs in at least one brain region. **C** DEGs involved in social behaviour, *y* axis indicates the transcripts per million (TPM) of genes. **D** All differentially expressed glutamate receptors (GluRs) and glutamate receptor-interacting proteins (GRIPs) displayed a higher expression (dark blue) in no-interacting individuals. Heatmap were created based on TPM value of genes and scaled by row, including 27 GluRs [22 ionotropic GluRs (iGluRs): 4 AMPAs, 1 GRID, 7 Kainates, 10 NMDAs; 5 metabotropic GluRs (mGluRs): 2 Group1 genes, 3 Group2 genes] and two GRIPs (GRIP1, GRIP2)

To evaluate molecular signals related to cleaning interactions in the genome of dedicated cleaner *L. dimidiatus*, transcriptomic data were compared between interacting and non-interacting *L. dimidiatus* individuals, which revealed 2735, 1582 and 512 differentially expressed genes (DEGs, Additional file 1: Table S11) in the forebrain (FB), hindbrain (HB) and midbrain (MB), respectively. The majority of genes were reduced in expression in interacting *L. dimidiatus* (Fig. 5A), with 65, 70 and 61% downregulated DEGs in FB, HB and MB when compared to non-interacting *L. dimidiatus* individuals. A total of 4004 DEGs were detected in at least one of three brain regions, of which 56.9% (2,278 DEGs) exhibited a reduced expression across all regions of *L. dimidiatus* during cleaning interactions.

The number of DEGs with reduced expression was even more remarkable in signal transductions especially in the sensory perception of touch and glutamate transmission (Fig. 5B, Additional file 1: Table S12), of which 100% (23 of 23 genes) and 93.5% (43 of 46 genes) have a decreased expression during cleaning. In addition, the majority of DEGs related to behaviours, such as locomotory exploration behaviour (93.3%, 28 of 30 genes) and social behaviour (84.8%, 56 of 66 genes), were also downregulated in interacting *L. dimidiatus* individuals. Among 66 DEGs underlying social behaviour (Additional file 1: Table S13), two glutamate receptors (*GRIN1*, *GRID1*) and three glutamate decarboxylases (2 *GAD1*, *GAD2*) were downregulated in *L. dimidiatus* individuals during cleaning (Fig. 5C). Moreover, twenty-seven glutamate receptors (GluRs) and two glutamate receptor-interacting proteins (GRIPs) were significantly expressed between interacting and non-interacting *L. dimidiatus* individuals, and all these GluRs and GRIPs were lower expressed in interacting *L. dimidiatus* individuals (Fig. 5D, Additional file 1: Table S14). In addition, we also examined expression levels in *L. dimidiatus* for olfactory receptors (Additional file 2: Fig. S8) and vision opsin genes (Additional file 2: Fig. S9), which displayed a low expression with no significant differences between interacting and non-interacting *L. dimidiatus* individuals. Similar results were also found in protocadherins (Additional file 2: Fig. S10) and pathogen recognition receptors (PRRs, Additional file 2: Fig. S11) apart from a protocadherin (protocadherin alpha-C2: *PCDC2*) and a PRR (Toll-like receptor 7: TLR7) with a significantly different expression when interacting.

## Discussion

Cleaning behaviour is a critical foraging strategy of cleaner fish with tremendous importance in maintaining a healthy reef ecosystem and aquaculture systems [1]. A chromosome-scale reference genome of *L. dimidiatus*, a prime candidate with dedicated cleaning behaviour, allowed for novel insights into the molecular adaptations of this important and fascinating behaviour. A variety of substantial gene family contractions were detected in *L. dimidiatus*, with positively selected genes and transcriptional changes in key functions. We further detected convergent evolution in key functions between dedicated and facultative cleaners allowing to pinpoint the genetic and functional basis of this complex cleaning behaviour.

### Sensory perception of vision and olfaction

Animals commonly use olfaction and vision for foraging and to avoid predators [36, 37]. As the cleaner fish mainly eats parasites which appear as small dark dots on the surface of clients [29], it can be assumed that cleaner fish would have good olfaction or vision. Surprisingly, the number of vision opsin genes is similar across dedicated, facultative and non-cleaners showing no evidence of vision-related changes involved in cleaning behaviour. However, the olfactory receptors (ORs) exhibited distinctive massive contractions in the dedicated cleaner *L. dimidiatus*. These ORs contractions may reveal a decreased importance of olfaction in dedicated cleaners compared to other facultative and non-cleaner fish species. Fish species which feed on a variety of food sources may rely heavily on olfaction to detect different foods [38, 39]. However, the dedicated cleaner fish mainly depends on advertising their services through dancing and conspicuous colourful stripes to gain access to client fish [1, 10–12, 40]. Since cleaners inhabit areas with generally good visibility and client fishes directly approach cleaners asking for service, the requirement for olfaction might be reduced in cleaning interactions. In fact, the contraction of ORs can be linked to dietary transitions [41], and the observed difference between facultative and dedicated cleaners in the number of ORs may be a result of specialization in cleaning as facultative species only get half of their food from cleaning interactions [4]. In addition, olfaction is essential for fish species in predator recognition as well as alarm cues from conspecifics warning of predation danger [42, 43]. However, predation on cleaners especially the dedicated cleaners have been rarely observed during cleaning even when they serve large predators [4, 44, 45]. Due to high specialization and dependency on cleaning, predator avoidances are not as key to survival as for the vast majority of other coral reef fishes. As such, olfactory receptors may have lost importance leading to the observed contractions in ORs.

### Sensory perception of touch and taste

In addition to vision and olfaction, fish can also depend on tactile or taste sensory information to find and ingest food [46, 47]. Tactile stimuli also play an important role in the exploratory and social behaviour of fish through their tactile organs such as fins, barbels and dermal teeth [47–49]. Cleaner wrasses *L. dimidiatus* developed the capacity to use tactile stimulation (“massage”-like behaviour, where cleaners rub the body surface of client with their pectoral fins) as a pre-conflict managing strategy after dishonesty and as a client stress managing strategy [50, 51]. This pre-conflict strategy only evolved in cleaners from the clade labrichthyines (only *L. dimidiatus* in our studies species). These cleaners prefer to eat mucus instead of parasites (i.e., being dishonest), enhancing the need to employ pre-conflict strategies such as tactile stimulation to maintain interactions [21]. Notably, the dedicated cleaner *L. dimidiatus* displayed twenty-three genes involved in sensory perception of touch with significantly increased expression when not interacting and decreased expression in interacting *L. dimidiatus*, indicating a possible preparedness for interaction. Of these twenty-three touch sensory perception genes, a glutamate receptor (*GRIN1*) and seven a-type potassium channel genes are known for extracting information from sensory inputs [52] and pain-sensing [53] respectively. Besides, feeding behaviour is also affected by their taste-discrimination capacity [54]. The dedicated and facultative cleaners were detected with convergent evolving signals in a taste receptor *TAS1R3*, which is responsible for the sensory perception of sweet taste [55] and may thus contribute to differences in the food they ingest and even cause dietary transitions between cleaners and non-cleaners. Hence, dedicated cleaner fish may pre-heighten the touch sensory perception for cleaning interactions and food ingestions by their evolved taste sensory system.

### Morphological changes

Low-displacement and fast jaw movements enable cleaner fish to rapidly and dexterously touch clients using their subterminal mouths for the removal ectoparasites on clients [1, 14]. Bone morphogenetic proteins (BMPs) are pivotal morphogenetic signals related to the formation of bone and cartilage in fish [56, 57]. Here we found that the dedicated and facultative cleaners have a convergent evolution in *BMP10*, which was also seen to induce jaw deformity in golden pompano larvae when this gene was lowly expressed [58]. Moreover, three other genes (*LRRC17*, *CHAD*, *THBS4B*) involved in bone development also exhibited convergences among all cleaners in our study. For instance, *LRRC17* [59] and *CHAD* [60] may contribute to effective amelioration of bone loss. Highly expressed *THBS4B* in articular chondrocytes is essential for maintaining articular cartilage integrity [61]. Furthermore, the dedicated cleaner displayed positive selection in *GDF2* (also known as *BMP9*), which can modulate dentinogenesis and tooth development [62, 63]. Therefore, the amino acid convergences observed in four genes across cleaners may play a role in the differences in jaw structures between cleaners (dedicated and facultative cleaners) and non-cleaners [64]. Additionally, *GDF2* could potentially contribute to further jaw divergence specifically in dedicated cleaners.

### Enhanced immune defense

Parasites have been widely documented with negative impacts on the growth, survival and reproductive success of their fish hosts [65, 66]. Therefore, the cleaning benefits appear to be obvious; clients benefit from the removal of parasites and cleaners benefit with a source of food. However, the benefit probably comes with a cost for cleaners due to the transmission of the ectoparasites onto themselves from the clients during close-contact cleaning interactions [16, 17]. In addition, parasite infection pressure may be even higher around cleaning stations due to the occurrence of clients with a higher risk of parasitic infection [67]. Hence, detecting the pathogens and initiating an innate immune defense is critical for cleaners to refrain from the parasitism risk. We found that *NLRC3* (NLR family CARD domain-containing protein 3), a pathogen recognition receptor, exhibited substantial contractions in the dedicated cleaner *L. dimidiatus* when compared to its closely relative facultative and non-cleaner. *NLRC3* can attenuate toll-like receptor signalling [68] and negatively regulate innate immune signalling induced by interferon genes [69], which could be helpful for *L. dimidiatus* in adjusting to elevated parasite loads. Since dedicated cleaners engage in more cleaning interactions than facultative and non-cleaners, dedicated cleaners are exposed to a higher parasitism risk; thus, a strong selective pressure to evolve a stronger innate immunity to avoid the negative impacts from parasites might be present.

### Specializations in neural signalling transduction

As a dedicated cleaner fish that prefers to eat mucus (cheat), *L. dimidiatus* evolved a set of highly sophisticated cognition and decision-rules techniques to manipulate their clients. This highly sophisticated cognition may require a complex nervous system. However, the dedicated cleaner *L. dimidiatus* exhibited contractions in protocadherins, compared to facultative cleaner and non-cleaner. Protocadherins are homophilic cell adhesion molecules required for neuronal development as well as synaptic specificity [32]. Substantial expansions of protocadherins have been reported to play a critical role in the formation of large and complex nervous systems of octopus [70]. Hence, a reduced number of protocadherins may lead to a simplified or more specialized nervous system in dedicated cleaner *L. dimidiatus*. We further observed a divergence of glutamatergic transmission genes between cleaners (dedicated and facultative cleaners) and non-cleaners that might contribute to the specializations in neural signalling transduction of cleaners. As the most abundant excitatory neurotransmitter in the brain, glutamate plays a major role in learning and memory [71, 72], which can consolidate taste-recognition memory [73, 74]. In particular, dedicated cleaner *L. dimidiatus* showed positive selections in a glutamate receptor (*GRIA3*) and *ADCY1*, both of which are crucial for the release of glutamate [75]. Furthermore, we found that twenty-nine glutamate receptors with significant expression differences between interacting and non-interacting *L. dimidiatus*, and twenty-seven glutamate receptors have significantly increased expression when not interacting as possible preparedness for interaction. Thus, protocadherin reductions and evolved glutamatergic transmission genes may allow for the dedicated cleaner *L. dimidiatus* to have evolved specializations in neural signalling transduction for social learning and cognition.

## Conclusions

Cleaner wrasses specialized their behaviour and morphology to obtain food from social interactions. Here we produced a high-quality chromosome-scale genome of *L. dimidiatus* to investigate molecular adaptations underlying the dedicated cleaning behaviour by the comparison with facultative and non-cleaner fishes. *L. dimidiatus* experienced substantial contractions in olfactory receptors (ORs), innate immune receptors and protocadherins. Meanwhile, cleaners (dedicated and facultative) displayed divergences in genes associated with taste-discriminate, glutamatergic transmission and bone formation. In addition, the dedicated cleaner *L. dimidiatus* is more likely to exhibit elevated gene expression in brain regions when not interacting as a possible preparedness for cleaning, especially notable for genes related to touch sensory perception and glutamatergic transmission. Therefore, we conclude that, compared with facultative and non-cleaners, dedicated cleaning behaviour have a higher dependency on touch and taste than olfaction and vision for sensory perception, a distinct jaw for food ingestion, an enhanced immune response to lessen the impact of parasitism, and a specialized nervous system for neural signal transduction. Our results provide novel and important insights into molecular adaptations underlying dedicated cleaning behaviour.

## Methods

### High molecular weight DNA extraction and PacBio sequencing

A single blue-streak cleaner wrasse fish (6.8 cm), obtained from a local store, was used to obtain sample tissues. To obtain sufficient high-quality genomic DNA for the whole genome sequencing, brain (23 mg), gills (30.2 mg) and muscle (367.1 mg) were aseptically dissected out, snap-frozen in liquid nitrogen for at least 1 h and then stored at − 80 °C. DNA were extracted from muscle tissues according to the Qiagen Genomic DNA Handbook (Qiagen) and Genomic-Tip 500/G (Qiagen) procedure. The quality of the DNA was checked by agarose gel electrophoresis, and excellent integrity DNA molecules were observed.

### HiFi PacBio library preparation and sequencing

DNA purity was assessed on a NanoDrop NP-1000 spectrophotometer (NanoDrop Technologies), DNA concentration was measured with a Qubit dsDNA high-sensitivity assay and DNA size was validated by pulsed-field gel electrophoresis (PFGE). Ten micrograms of DNA was sheared to the appropriate size range (10–30 kb) using a Covaris g-TUBE for the construction of PacBio HiFi sequencing libraries, followed by bead purification with PB Beads (PacBio). Sequencing libraries were constructed following the manufacturer’s protocol using a SMRTbell Express Template Prep Kit 2.0. Libraries were quantified using the Qubit dsDNA high-sensitivity assay, and size was checked on a Femto Pulse System (Agilent). Sequencing was performed on PacBio Sequel II systems in circular consensus sequencing (CCS) mode for 30 h.

### Omni-C library preparation and sequencing

For each Omni-C library, chromatin was fixed in place with formaldehyde in the nucleus and then extracted. Fixed chromatin was digested with DNase I, chromatin ends were repaired and ligated to a biotinylated bridge adapter followed by proximity ligation of adapter containing ends. After proximity ligation, crosslinks were reversed and the DNA purified. Purified DNA was treated to remove biotin that was not internal to ligated fragments. Sequencing libraries were generated using NEBNext Ultra enzymes and Illumina-compatible adapters. Biotin-containing fragments were isolated using streptavidin beads before PCR enrichment of each library. The library was sequenced on an Illumina HiSeqX platform to produce ~ 30 × sequence coverage. Then HiRise used (See read-pair above) MQ > 50 reads for scaffolding.

### De novogenome assembly and scaffolding

22.6 gigabase-pairs of PacBio CCS reads were used as an input to Hifiasm v0.15.4-r347 [76] with default parameters. The initial assembly was mapped against the nucleotide sequence database by Blastn, and the results were used as input for blobtools v1.1.1 [77] to remove the scaffolds that were identified as possible contamination in the assembly. Finally, purge_dups v1.2.5 [78] was used to identify and remove both haplotigs and heterozygous overlaps.

The de novo assembly and Omni-C library reads were used as input for HiRise, a software pipeline designed specifically for using proximity ligation data to scaffold genome assemblies [79]. Omni-C sequences were aligned to the draft input assembly using bwa (https://github.com/lh3/bwa). The read pairs mapped within draft scaffolds were applied to produce a likelihood model for genomic distance between scaffolds for joining. Based on Actinopterygii odb9 database comprising 4584 single-copy orthologs, the completeness of genome assembly was assessed by BUSCO v3.1.0 [28] using the predicted genes and whole genome assembly respectively.

### Mitochondrial genome assembly

Mitochondrial DNA is a useful and particularly popular marker for molecular ecology, population genetics and phylogenetic studies [80], while traditional genome assembly software can hardly assemble complete mitogenomes [81]. With *Notolabrus celidotus* mitochondrial genome as the reference and a readpool by sampling 10% Ominc reads, we assembled the mitochondrial genome of *L. dimidiatus* using MITObim v1.9.1 [80] by 30 iterations “-start 1 -end 30”, and MITObim reached a stationary read number after 30 iterations. The resulting assembly was annotated for genes using MitoAnnotator [82]. To confirm the species of the sampled *L. dimidiatus* individual, we constructed a phylogeny based on Cytochrome c oxidase subunit I gene (COI), 12S and 16S of 10 Labridae species (*L. dimidiatus*, the individual in our study and an individual from NCBI; *L. phthirophagus*; *L. bicolor*; *L. pectoralis*; *L. rubrolabiatus*; *Symphodus melops*; *Labrus bergylta*; *Thalassoma bifasciatum*; *Cheilinus undulatus*; *Notolabrus celidotus*) and three other species (Medaka, *Oryzias latipes*; Fugu, *Takifugu rubripes*; Stickleback, *Gasterosteus aculeatus*; Zebrafish, *Danio rerio*; Platyfish, *Xiphophorus maculatus*; Spotted gar, *Lepisosteus oculatus*) (Additional file 1: Table S15).

### RNA sequencing and gene expression analyses

For genome annotation and expression analyses, fore-, mid- and hindbrain tissue were obtained from 30 adult individuals of *L. dimidiatus* for RNA sequencing. The specimens were collected in the Maldives Islands and transported to the aquatic facilities of Laboratorio Maritimo da Guia in Cascais, Portugal by TMC-Iberia, further details can be found in [15] and Ramirez-Calero et al*.* (2023, unpublished). For the experiment evaluating transcriptional changes for cleaning behaviour, *L. dimidiatus* (*N* = 6) or clients (*N* = 6) were kept alone in the observation tank (control) as no-interaction treatment, while one cleaner (*N* = 6) and one client (*N* = 6) were kept together in the observation tank as the interaction treatment, allowing them to have close contact. Their interactions were filmed for 40 min since it is documented that this is the time frame in which neurohormones, and peptides are activated during cleaner interactions [83]. At the end of the observation period, three separated regions (forebrain, midbrain, hindbrain) of the brain were immediately dissected out for each *L. dimidiatus* individuals for RNA sequencing. The details of sequencing and reads quality control can be found in [15].

To identify gene expression differences of three brain tissues between non-interacting and interacting *L. dimidiatus* individuals, high-quality reads were mapped against the *L. dimidiatus* genome assembled in this study with HISAT2 v2.1.0 [84]. Read number matrices of all genes were generated using FeatureCounts v2.0.0 [85] and then were used as input for DEseq2 [86] to estimate the differential expressed genes (DEGs) between non-interacting and interacting *L. dimidiatus* individuals. DEGs should display with an FDR adjusted *P* value ≤ 0.05 and the average of the normalized count values (basemean) ≥ 10 as well as the effect size (Log2FoldChange) ≥ 0.3. Functional enrichment analyses were performed for all gene sets of interest by comparison with the whole gene data set with Fisher’s exact test in OmicsBox v2.0.29. Functions were accepted as significantly enriched with a false discovery rate (FDR) Padj < 0.05 and reduced to most specific terms.

### Repeat elements and gene annotation

To compare the genomes of fish with cleaning behaviours, we obtained the sequences of seven other Labridae fish species, including five facultative cleaner (*Thalassoma bifasciatum*, GenBank: RPOG00000000.1; *Symphodus melops*, GenBank: MWVA00000000.1; *Tautogolabrus adspersus*, GCA_020745685.1; *Semicossyphus pulcher*, GCA_022749685.1; *Labrus bergylta*, GenBank: FKLU00000000.1) and two non-cleaner (*Cheilinus undulatus*, GenBank: GCA_018320785.1; *Notolabrus celidotus*, GenBank: GCA_009762535.1). Their assemblies and *L. dimidiatus* assembly were annotated by searching repeated elements using RepeatModeler v2.0.2 [87]. The assembled genome sequences were used as input to generate a de novo library for RepeatModeler. Then, RepeatModeler was run (-LTRStruct) by combining the results of LTR structural discovery pipeline (LTR_Harvest and LTR_retreiver) and RepeatScout/RECON pipeline. Consensus sequences of the repeated elements were subsequently used to mask repeats in the assembly using RepeatMasker v4.1.2-p1 [88].

The soft-masked genome of *L. dimidiatus* was then predicted by BRAKER v2.1.6. [89–97], BRAKER ran twice respectively using OrthoDB proteins (–softmasking; –AUGUSTUS_ab_initio; –gff3; –prot_seq) and RNA-seq data on the fore-, mid- and hindbrain of 30 adult *L. dimidiatus* individuals (–softmasking; –AUGUSTUS_ab_initio; –gff3; –UTR = on; –bam). The two results obtained were combined using TSEBRA v1.0.3 [98] with default parameters. For the five Labridae species, we also re-predicted their protein-coding genes using OrthoDB proteins. The longest transcripts of all predicted coding genes were selected for homology annotation with the proteins from Swiss-Prot and vertebrate_other by diamond v0.9.24.125 [93].

### Estimation of gene family expansion and contraction

Gene family expansion or contraction is thought to be an important driving force to evolutionary novelties, such as cleaning behaviour. To examine and compare the gene family dynamics, in addition to the eight Labridae fish species, the protein sequences of another six reference fish species with high-quality genome were downloaded by BioMart from Ensembl (Japanese Medaka, *Oryzias latipes* ASM223467v1; Fugu, *Takifugu rubripes* fTakRub1.2; Stickleback, *Gasterosteus aculeatus* BROAD S1; Zebrafish, *Danio rerio* GRCz11; Platyfish, *Xiphophorus maculatus* X_maculatus-5.0-male; Spotted gar, *Lepisosteus oculatus* LepOcu1). For all of 14 fish species, the longest protein per gene was selected, the genes with protein sequences shorted than 30 amino acids or have early stop codons in the coding regions were removed. The protein sequences of remaining genes were applied to detect orthologous genes using the default parameters in OrthoFinder v2.3.3. The 13,861 of 22,528 orthogroups including at least one gene from zebrafish and genes among at least four reference fish species were deemed as conserved gene families. And the matrix including gene numbers of these 13,861 conserved gene families were parsed to identify gene family dynamics by CAFE v4.2.1 [99].

### Phylogenetic analysis and divergence time estimation

The 2915 one-to-one orthologous genes of all 14 fish species were selected to construct the phylogenetic tree. MUSCLE v3.8.31 [100] was applied to align the protein sequences; regions with poor quality were trimmed using trimAl v1.4.rev22 [101] by the following parameters: -gt 0.8 -st 0.001 -cons 60. Then the sequences of proteins were concatenated for phylogeny detection by RaxML v8.2.11 [101]. The parameter PROTGAMMAAUTO was used to select the optimal amino acid substitution model. Then MCMCtree [33] was used to investigate the divergence time under a relaxed-clock model (correlated molecular clock) with approximate likelihood calculation. First, the coding nucleotide sequences were aligned by MUSCLE v3.8.31 [100] and trimmed by trimAl v1.4.rev22 [101] with “-gt 0.9 -st 0.001 -cons 60”. Based on the above resulting phylogenetic tree and nucleotide sequences, substitution rate was roughly estimated by baseml under the general time reversible (GTR) model suggested by jModelTest [102]. Then MCMCtree was run for the first time to estimate the gradient and Hessian, the output (out.BV) was used for the second running of MCMCTree to perform approximate likelihood calculations. The final Markov chain Monte Carlo process was run by “burnin = 50000; sampfreq = 100; nsample = 2000000”. We set two fossil calibrations: *O. latipes*–*T. nigroviridis* (~ 96.9–150.9 Ma), *D. rerio*–*G. aculeatus* (~ 149.85–165.2 Ma) and a time for the root (< 700 Ma).

### Olfactory receptors

Due to the potential involvement of olfaction and vision in the interaction behaviours displayed by *L. dimidiatus*, we compared the olfactory and vision genes among species. The olfactory receptor (OR) genes [103] were downloaded as query sequences and mapped to genome of fourteen fish species by tblastn with “1e − 5”. Solar (in-house software, version 0.9.6) was used to join the high-scoring segment pairs (HSPs) between each pair of protein mapping results if overlaps were detected in the mapping regions of two hits. The best alignments to the same mapping regions with lengths longer than 200 in the genome were kept. Subsequently, GeneWise v2-4–1 [104] was applied to map the protein sequences to genome regions which extended 280 bp upstream and downstream of their mapping genome region. If a genomic nucleotide region were mapped by multiple query proteins, the one with highest GeneWise score were kept and the predicted protein sequence was annotated by Swiss-Prot. The genes with description including keywords “olfactory” or “odorant” were retained as putative OR genes. The hmmscan was applied to search against Pfam database to identify the domain with the highest score for each putative OR gene. The putative OR genes with a coding sequence from start codon to a stop codon were considered as intact OR genes, which were BLASTP to OR query sequences for OR subfamilies classification. These intact OR genes were aligned using MUSCLE, and the alignments were used to construct a phylogenetic tree by FastTree2 for the verification and correction of these putative OR genes. For the final maximum likelihood phylogenetic tree, the following eight non-OR genes [105] were used as the outgroup sequences: alpha-1A adrenergic receptor isoform 1 (NP_000671.2), beta-1 adrenergic receptor (NP_000675.1), adenosine receptor A2b (NP_000667.1), histamine H2 receptor isoform 2 (NP_071640.1), 5-hydroxytryptamine receptor 1B (NP_000854.1), 5-hydroxytryptamine receptor 1F (NP_000857.1), 5-hydroxytryptamine receptor 6 (NP_000862.1), galanin receptor type 1 (NP_001471.2) and somatostatin receptor type 4 (NP_001043.2). The putative OR gene sequences of eight Labridae fish species and eight non-OR genes were aligned by MUSCLE and trimmed by trimAl v1.4.rev22 [101] by “ -gt 0.8 -st 0.001 -cons 60,” the resulting alignments were used to construct the maximum likelihood phylogenetic tree by RaxML v8.2.11 [101] with PROTGAMMAAUTO to select the optimal amino acid substitution model.

### Opsin genes

The opsin genes (RH1: rhodopsin; RH2: middle-wavelength-sensitive opsin rhodopsin 2; LWS: long-wavelength-sensitive opsin; SWS1: short-wavelength-sensitive opsin 1; SWS2: short-wavelength-sensitive opsin 2) of spotted gar, zebrafish, medaka, platyfish, fugu and stickleback were used as query protein sequences. And these query protein sequences were mapped to genome of fourteen fish species by tblastn with “1e − 5”. Solar (in-house software, version 0.9.6) was used to join the high-scoring segment pairs (HSPs) between each pair of protein mapping results if overlaps were detected in the mapping regions of two hits. The best alignments to the same mapping regions with lengths longer than 50 in the genome were kept. Subsequently, Genewise was applied to map the protein sequences to genome regions which extended double of the query proteins length along upstream and downstream of their mapping genome region. If a genomic nucleotide region were mapped by multiple query proteins, the one with highest Genewise score were kept and its predicted protein sequences was annotated by Swiss-Prot; the genes were annotated as opsin genes which covered at least 70% and with e-value < 1e − 20 of the corresponding proteins in Swiss-Prot were retained as putative opsin genes, which were aligned by MUSCLE, and trimmed by trimAl v1.4.rev22 [101] by “ -gt 0.8 -st 0.001 -cons 60” to construct the maximum likelihood phylogenetic tree by RaxML v8.2.11 [101] with PROTGAMMAAUTO to select the optimal amino acid substitution model.

### Protocadherin, innate immunity family and glutamate receptors

Using the same method as opsin gene identification, gene family members for protocadherin, innate immunity families and glutamate receptors were identified in fourteen fish species from the phylogenetic tree (Fig. 1A). Protocadherin alpha and gamma genes from Swiss-Prot were used as query proteins. Based on this, a domain search against the Pfam database was conducted using hmmscan, and genes with over six extracellular cadherin domains were considered putative protocadherin alpha and gamma genes [70]. These genes were used to build a phylogenetic tree following the opsin gene identification method.

For innate immunity families, protein sequences of toll-like receptors (TLRs), RIG-like receptors (RLRs) and NOD-like receptors (NLRs) from six reference fish species (spotted gar, zebrafish, medaka, platyfish, fugu, stickleback) were used as queries. Predicted genes covering at least 70% and having an e-value < 1e − 20 were considered innate immunity family members and used for phylogenetic tree construction.

For glutamate receptors, protein sequences from six reference fish species (spotted gar, zebrafish, medaka, platyfish, fugu, stickleback) were used. The identified ionotropic glutamate receptors (iGluRs) with the SYTANLAAF motif [106] and metabotropic glutamate receptors (mGluRs) with seven transmembrane regions were retained [107].

### Evolutionary analysis

The orthogroups generated by OrthoFinder v2.3.3 [108] were divided into sub-orthogroups by Possvm v1.1 [109]. The genes within sub-orthogroups were classified into different groups according to their gene names, and only the groups containing all of the 14 species were kept. For species with more than two genes in the subgroup, the one with highest blast score was kept. The genes in the 6929 subgroups were considered as orthologous genes for the following evolutionary analysis. The protein sequences of orthologous genes in the subgroup were aligned by Clustal Omega-1.2.4 (-t Protein; –outfmt = fa) [110]. The protein alignments and corresponding coding nucleotide sequences were used as input for pal2nal v.14 [111] to construct the protein–cDNA sequence pairs, and poorly aligned positions and divergent regions of cDNA were removed by Gblocks v.0.91b (options: -b4, 10; -b5, n; –b3, 5; –t = c) [112].

To explore the possibility of cleaners evolving more rapidly compared to non-cleaners, we used the free-ratio model of codeml in PAML v.4.9 [33] to estimate the dN/dS (nonsynonymous-synonymous substitution ratio) along each lineage based on all 6929 orthologous genes. The results of each lineages under study were curated to reduce errors [113] by removing genes with any one of the following values: dS > 1, N > sequence length, N + S > sequence length by 50 or more bp and N*dN or S*dS < 1. The final gene number to estimate evolutionary rate were 5215 (*L. dimidiatus*), 5342 (*T. bifasciatum*), 5563 (*S. melops*), 4655 (*T. adspersus*), 5789 (*S. pulcher*), 5654 (*L. bergylta*), 5961 (*C. undulatus*), 5972 (*N. celidotus*), 6256 (Japanese Medaka: *O. latipes*), 6264 (Fugu: *T. rubripes*), 6259 (Stickleback: *G. aculeatus*), 4514 (Zebrafish: *D. rerio*), 6227 (Platyfish: *X. maculatus*) and 2219 (Spotted gar: *L. oculatus*). The mean dN/dS values of the qualified orthologous genes in the eight Labridae fish species were employed to compare their respective evolutionary rates.

To identify genes evolving under positive selection, the branch-site model of codeml in PAML v.4.9 [33] was applied to investigate the positively selected genes (PSGs) for *L. dimidiatus* based on the species tree. The terminal branch of *L. dimidiatus* was set as the foreground branch, a likelihood ratio test (LRT) was used to estimate whether the branch-site model containing positively selected codons (omega > 1) fits better than the null model including neutral selection or negative selection (omega ≤ 1). Chi-square statistics wrapped in PAML were performed to estimate the *P* values for model comparison, and the *P* values were corrected by the false discovery rate (FDR) using R version 3.6.3. Only the genes with an LRT FDR < 0.05 and containing codon sites with a posterior probability of positive selection over 0.95 by the Bayes empirical Bayes (BEB) method were treated as PSGs. To account for the impact of multi-nucleotide substitutions on natural selection detection [114], we also used BUSTED-MH method implemented in HYPHY v2.5.51 [34] to detect positively selected genes in *L. dimidiatus* with parameters “hyphy busted –alignment –tree –multiple-hits Double + Triple –starting-points 5 –branches”. With *L. dimidiatus* as the foreground branch, the LRT *P* value for episodic diversifying positive selection was corrected for FDR using R version 3.6.3. Genes with an LRT FDR < 0.05 were considered positively selected. Only genes detected by both PAML and HYPHY were deemed as the final positively selected genes.

To unveil convergent molecular evolution at the amino acid variation level underlying the cleaning behaviour in fish, we applied a method in Xu et al. [35] for detecting convergence at conservative sites (CCS) where all non-cleaner species (two non-cleaner Labridae fish and six reference species) shared the same amino acid. The noise estimation for the CCS method involved the following steps: (1) Sequence simulation: the amino acid sequences of 6,353 orthologous genes (7,720,731 amino acids) were concatenated to estimate branch lengths, amino acid frequencies and the best shape parameter for variable rates among sites (alpha) using codeml in PAML [33] with the JTT + gamma model. Using these parameters, we simulated amino acid sequences of the same length (7,720,731 amino acids) as the real data set with the evolver tool from PAML; (2) Ancestral state inference: the ancestral amino acid sequences were inferred using the empirical Bayesian ancestral reconstruction in codeml with the same parameters as the sequence simulation; (3) Accuracy estimation: sites were considered as convergences across cleaner fish if they were shared by at least three cleaner species and differed from any non-cleaner species. Among these convergences, sites were classified as random convergences if more than two cleaners showed the same amino acid as non-cleaners, and as false convergences if the ancestral amino acids of all non-cleaners (excluding the spotted gar *Lepisosteus oculatus*) were different with the spotted gar. The ancestral accuracy was estimated by comparing the amino acids of the spotted gar, and the ancestor of all non-cleaners (excluding spotted gar). The inferences of amino acids in ancestor were correct if they were identical with spotted gar; if not, the inferences were incorrect. To remove the random convergent sites, only sites in six cleaners that are not consistent with the CCS in all non-cleaners were considered as potential convergent evolution across six cleaners.

### Supplementary Information

**Additional file 1: Table S1.** Metrics for *Labroides dimidiatus* assembly. **Table S2**. The length and number of coding genes in *Labroides dimidiatus* genome. **Table S3.** BUSCO results (The lineage dataset is: actinopterygii_odb9) using predicted genes in genome of each species as input. **Table S4.** Percentage of different category of repeat elements in *Labroides dimidiatus* genome. **Table S5.** Dynamics of Gene family size of *Labroides dimidiatus*. **Table S6.** The number of predicted intact olfactory receptor (OR) in all of 14 species in our study. **Table S7.** The number of glutamate receptors identified among eight Labridae fishes. **Table S8.** Positively selected genes (41 genes) of *Labroides dimidiatus* detected by both PAML and HYPHY. **Table S9.** Noise Estimated by Simulations—Number of sites of random/false convergence. **Table S10.** Genes with convergent substitutions in dedicated cleaner (*Labroides dimidiatus*) and facultative cleaners (*Symphodus melops*, *Semicossyphus pulcher*, *Tautogolabrus adspersus*, *Thalassoma bifasciatum*, *Labrus bergylta*). **Table S11.** Differentially expressed genes (DEGs) between non-interaction (solo) and interaction (inte) groups of *Labroides dimidiatus*. **Table S12.** GO enrichment of all 4,004 differentially expressed genes (DEGs) between non-interaction (solo) and interaction (inte) groups of *Labroides dimidiatus*. **Table S13.** Genes related to social behavior of differentially expressed genes (DEGs) between non-interaction (solo) and interaction (inte) groups of *Labroides dimidiatus*. **Table S14.** Differentially expressed glutamate receptors and glutamate receptor-interacting proteins (from top to bottom in heatmap) between non-interaction (solo) and interaction (inte) groups of *Labroides dimidiatus*. **Table S15.** The sequences of 12S, 16S and COI used in the construction of phylogenetic tree to confirm the species of the sampled *Labroides dimidiatus* individual.

**Additional file 2: Figure S1.** Gene number along the whole genome. **Figure S2.** The divergence time of the Labridae lineage based on 2,915 single-copy gene families that only have one gene copy for all species. **Figure S3.** Phylogenetic tree of olfactory receptors (ORs) subfamily ζ gene sequences with 100 bootstraps and the non-ORs as the outgroup. **Figure S4.** Visual opsin genes among the dedicated cleaner *L. dimidiatus*, five facultative and two non-cleaners in the fish family Labridae. **Figure S5.** Phylogenetic tree of NOD-like receptors (NLRs) with 100 bootstraps and rooting at the midpoint. **Figure S6.** Phylogenetic tree of protocadherin alpha gene sequences with 100 bootstraps and rooting at the midpoint. **Figure S7.** Phylogenetic tree of protocadherin gamma gene sequences with 100 bootstraps and rooting at the midpoint. **Figure S8.** Expression of olfactory receptors (ORs) in the three brain regions (forebrain: FB, hindbrain: HB, midbrain: MB) of interacting and non-interacting *L. dimidiatus* individuals. **Figure S9.** Expression of opsin genes in the three brain regions (forebrain: FB, hindbrain: HB, midbrain: MB) of interacting and non-interacting *L. dimidiatus* individuals. **Figure S10.** Expression of protocadherins α and γ genes in the three brain regions (forebrain: FB, hindbrain: HB, midbrain: MB) of interacting and non-interacting *L. dimidiatus* individuals. **Figure S11.** Expression of ten pathogen recognition receptors in the thsree brain regions (forebrain: FB, hindbrain: HB, midbrain: MB) of interacting and non-interacting *L. dimidiatus* individuals.

## Data Availability

Genomic sequences (HiFi PacBio and Omni-C) and the de novo genome assembly have been deposited in NCBI under BioProject PRJNA937036 (https://www.ncbi.nlm.nih.gov/bioproject/PRJNA937036) [115], the transcriptomic sequences can be retrieved under BioProject PRJNA726349 (https://www.ncbi.nlm.nih.gov/bioproject/PRJNA726349) [116].

## Abbreviations

Gb: Gigabase
Mb: Megabases
Mya: Million years ago
dN/dS: Nonsynonymous-synonymous substitution ratio
PRRs: Pathogen recognition receptors
RLRs: RIG-like receptors
NLRs: NOD-like receptors
TLRs: Toll-like receptors
TLR7: Toll-like receptor 7
NLRC3: NLR family CARD domain-containing protein 3
PCDA2: Protocadherin alpha-2
PCDGB: Protocadherin gamma-A11
PCDC2: Protocadherin alpha-C2
PSGs: Positively selected genes
GRIA3: Glutamate receptor 3
ADCY1: Adenylate cyclase type 1
GDF2: Growth/differentiation factor 2
BMP9: Bone morphogenetic protein 9
CCS: Convergence at conservative sites
CEGs: Convergent evolving genes
TAS1R3: Taste receptor type 1 member 3
BMP10: Bone morphogenetic protein
LRRC17: Leucine-rich repeat-containing protein 17
CHAD: Chondroadherin
THBS4B: Thrombospondin-4-B
DEGs: Differentially expressed genes
FB: Forebrain
HB: Hindbrain
MB: Midbrain
GluRs: Glutamate receptors
GRIPs: Glutamate receptor-interacting proteins
iGluRs: Ionotropic glutamate receptors
mGluRs: Metabotropic glutamate receptors
ORs: Olfactory receptors
BMPs: Bone morphogenetic proteins
HSPs: High-scoring segment pairs
RH1: Rhodopsin
RH2: Middle-wavelength-sensitive opsin rhodopsin 2
LWS: Long-wavelength-sensitive opsin
SWS1: Short-wavelength-sensitive opsin 1
SWS2: Short-wavelength-sensitive opsin 2
LRT: Likelihood ratio test
FDR: False discovery rate
BEB: Bayes empirical Bayes
