# Supplementary material for: Gene losses, parallel evolution and heightened expression confer adaptations to dedicated cleaning behaviour
Source: BMC Biol. 2023 Aug 23;21:180. doi: 10.1186/s12915-023-01682-3 (PMC10463495; doi:10.1186/s12915-023-01682-3)
Supplement: Supplementary file 2 — Additional file 2: Figure S1. Gene number along the whole genome. Figure S2. The divergence time of the Labridae lineage based on 2,915 single-copy gene families that only have one gene copy for all species. Figure S3. Phylogenetic tree of olfactory receptors (ORs) subfamily ζ gene sequences with 100 bootstraps and the non-ORs as the outgroup. Figure S4. Visual opsin genes among the dedicated cleaner L. dimidiatus, five facultative and two non-cleaners in the fish family Labridae. Figure S5. Phylogenetic tree of NOD-like receptors (NLRs) with 100 bootstraps and rooting at the midpoint. Figure S6. Phylogenetic tree of protocadherin alpha gene sequences with 100 bootstraps and rooting at the midpoint. Figure S7. Phylogenetic tree of protocadherin gamma gene sequences with 100 bootstraps and rooting at the midpoint. Figure S8. Expression of olfactory receptors (ORs) in the three brain regions (forebrain: FB, hindbrain: HB, midbrain: MB) of interacting and non-interacting L. dimidiatus individuals. Figure S9. Expression of opsin genes in the three brain regions (forebrain: FB, hindbrain: HB, midbrain: MB) of interacting and non-interacting L. dimidiatus individuals. Figure S10. Expression of protocadherins α and γ genes in the three brain regions (forebrain: FB, hindbrain: HB, midbrain: MB) of interacting and non-interacting L. dimidiatus individuals. Figure S11. Expression of ten pathogen recognition receptors in the thsree brain regions (forebrain: FB, hindbrain: HB, midbrain: MB) of interacting and non-interacting L. dimidiatus individuals. [file 12915_2023_1682_MOESM2_ESM.docx]

# Gene losses, parallel evolution and heightened expression confer adaptations to dedicated cleaning behaviour

Jingliang Kang^1^, Sandra Ramirez^1^, José Ricardo Paula^1,2,3^, Yifang Chen^4^, Celia Schunter^1,4*^

^1^ Swire Institute of Marine Science, School of Biological Science, The University of Hong Kong, Pokfulam, Hong Kong SAR

^2^ MARE—Marine and Environmental Sciences Centre & ARNET – Aquatic Research Network, Laboratório Marítimo da Guia, Faculdade de Ciências, Universidade de Lisboa, Av. Nossa Senhora do Cabo, 939, 2750-374 Cascais, Portugal;

^3^ Departamento de Biologia Animal, Faculdade de Ciências, Universidade de Lisboa, Campo Grande, 1749-016 Lisboa, Portugal

^4^ State Key Laboratory of Marine Pollution and Department of Chemistry, City University of Hong Kong, Hong Kong, SAR, China

Supplementary Materials

Supplementary Figure 1:


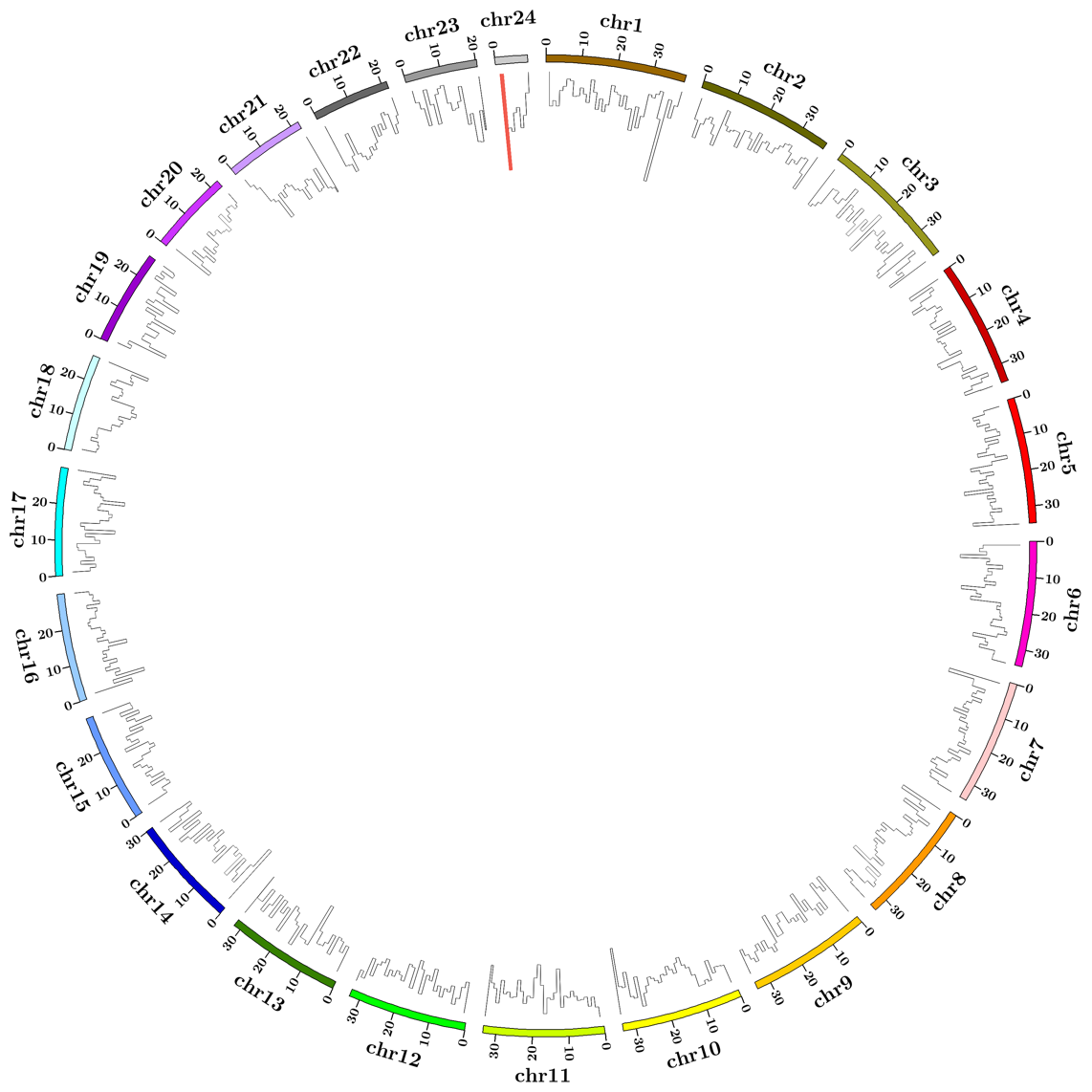


Figure S1. Gene number along the whole genome using a sliding window of 1 M start codon locating the genetic region; the red bar is a genetic region (chr24: 1000001-2000000) with a maximum gene start codon (136 genes).

Supplementary Figure 2:


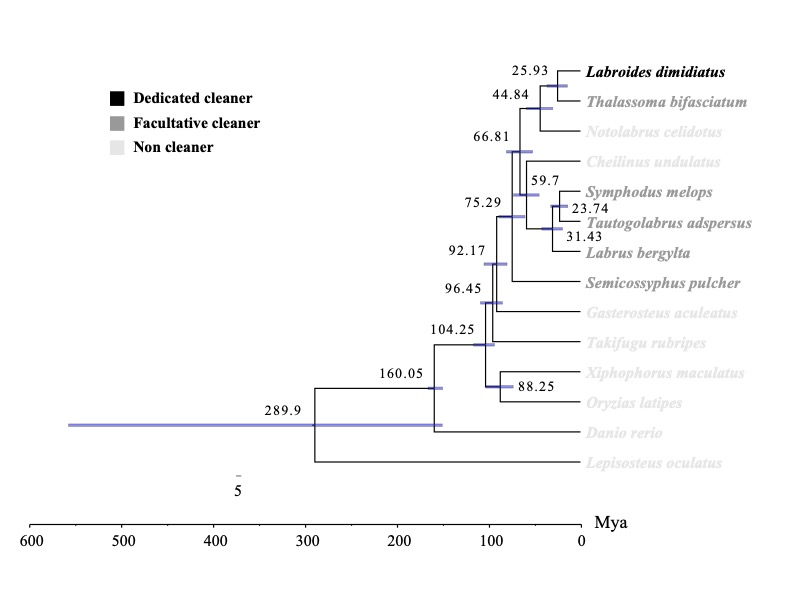


Figure S2. The divergence time of the Labridae lineage based on 2,915 single-copy gene families that only have one gene copy for all species. Branch lengths were calibrated using fossil records for the split of medaka/fugu and zebrafish/stickleback. Blue bars refer to the 95% confidence interval. Node labels represent the estimated mean time of node ages.

Supplementary Figure 3:

Figure S3. Phylogenetic tree of olfactory receptors (ORs) subfamily ζ gene sequences with 100 bootstraps and the non-ORs as the outgroup. *T. bifasciatum* and *N. celidotus* exhibited more ORs δ gene than *L. dimidiatus*. The leaf nodes with back, grey, and light grey square mean obligate cleaner, facultative cleaner, and non-cleaner. Only the internal nodes with bootstraps >=80 showed in the phylogenetic tree.

Supplementary Figure 4:

Figure S4. Visual opsin genes among the dedicated cleaner *L. dimidiatus*, five facultative and two non-cleaners in the fish family Labridae. Phylogenetic tree was constructed based on opsin gene sequences with 100 bootstraps and the pinopsins of spotted gar and fugu as the outgroup. The number of each opsin subfamilies showed according to the order as the species in the legend. The bold branches indicate the internal nodes with bootstraps >= 80.

Supplementary Figure 5:

Figure S5. Phylogenetic tree of NOD-like receptors (NLRs) with 100 bootstraps and rooting at the midpoint. The number of each NLR showed according to the order of the species in the legend. The bold branches indicate the internal nodes with bootstraps >= 80.

Supplementary Figure 6:

Figure S6. Phylogenetic tree of protocadherin alpha gene sequences with 100 bootstraps and rooting at the midpoint. The number of each opsin subfamily showed according to the order of the species in the legend. The bold branches indicate the internal nodes with bootstraps >= 80.

Supplementary Figure 7:

Figure S7. Phylogenetic tree of protocadherin gamma gene sequences with 100 bootstraps and rooting at the midpoint. The number of each opsin subfamily showed according to the order of the species in the legend. The bold branches indicate the internal nodes with bootstraps >= 80.

Supplementary Figure 8:

Figure S8. Expression of olfactory receptors (ORs) in the three brain regions (forebrain: FB, hindbrain: HB, midbrain: MB) of interacting and non-interacting *L. dimidiatus* individuals. 28 of 36 ORs in *L. dimidiatus* individuals were not expressed [transcripts per million (TPM) equals 0]. Almost ORs were few expressed, and ORs κ has the maximum expression.

Supplementary Figure 9:

Figure S9. Expression of opsin genes in the three brain regions (forebrain: FB, hindbrain: HB, midbrain: MB) of interacting and non-interacting *L. dimidiatus* individuals. *L. dimidiatus* displayed no difference in expression of opsin genes from no-interaction to interaction. The colour of gene mean the type (SWS1, SWS2, RH1, RH2, LWS) of vision opsins.

Supplementary Figure 10:

Figure S10. Expression of protocadherins α and γ genes in the three brain regions (forebrain: FB, hindbrain: HB, midbrain: MB) of interacting and non-interacting *L. dimidiatus* individuals, which have less than 80% (28 of 36) *L. dimidiatus* individuals with transcripts per million (TPM) < 1. Asterisk indicates the tissue whose gene showed differential expression between interacting and non-interacting *L. dimidiatus* individuals

Supplementary Figure 11:

Figure S11. Expression of ten pathogen recognition receptors (PRRs: two RLRs, four NLRs, four TLRs) in the three brain regions (forebrain: FB, hindbrain: HB, midbrain: MB) of interacting and non-interacting *L. dimidiatus* individuals. These pathogen recognition receptors were expressed in less than 80% (28 of 36) *L. dimidiatus* individuals with transcripts per million (TPM) < 1. Asterisk indicates the tissue whose gene showed differential expression between between interacting and non-interacting *L. dimidiatus* individuals.
